# Stream-based active learning for surgical AI

**DOI:** 10.1007/s11548-026-03718-9

**Published:** 2026-06-15

**Authors:** Gregor Just, Alexander C. Jenke, Antonia Kraneis, Johanna M. Brandenburg, André Schulze, Martin Wagner, Sebastian Bodenstedt, Stefanie Speidel

**Affiliations:** 1 Department of Translational Surgical Oncology, National Center for Tumor Diseases (NCT), NCT/UCC Dresden, a partnership between DKFZ, Faculty of Medicine and University Hospital Carl Gustav Carus, TUD Dresden University of Technology, and Helmholtz-Zentrum Dresden-Rossendorf (HZDR), Dresden, Germany; 2 Faculty of Medicine, University Hospital Carl Gustav Carus, TUD Dresden University of Technology, Dresden, Germany; 3 Cluster of Excellence-CeTI, TUD Dresden University of Technology, Dresden, Germany; 4 Department of Visceral, Thoracic and Vascular Surgery, Faculty of Medicine, University Hospital Carl Gustav Carus, TUD Dresden University of Technology, Dresden, Germany

**Keywords:** Active learning, Surgical AI, Model lifecycle, Intraoperative annotation

## Abstract

**Purpose:**

Continuous deployment in surgical AI promises adaptive models that evolve with clinical practice, yet its success critically depends on efficient annotation, balancing limited expert time with the need to identify and label only the most informative data to ensure sustained model improvement. We propose a Stream-based Active Learning pipeline where annotation and retraining extend beyond initial deployment, allowing models to adapt and mitigate distribution shifts. Collecting expert annotations during surgery presents both challenges and opportunities: selecting non-redundant, informative data improves generalization, while immediate integration of expert feedback can refine model predictions in real time.

**Methods:**

To enable online selection of informative training data during surgery, we build upon VeSSAL, a Stream-based Active Learning method motivated by model gradients, utilizing both model uncertainty and data diversity in its acquisition function. We improve the sample selection of Active Learning by including an Adversarial Autoencoder (AAE) loss component during training, normalizing the latent features.

**Results:**

Our enhanced version of VeSSAL achieves a stable labeling ratio with 12% standard deviation, while improving the macro F1 score by up to 7.7% after 15 surgeries over equidistant sampling and thresholding strategies of uncertainty and variance. We evaluate on a private dataset and Cholec80.

**Conclusions:**

We demonstrate that intraoperative, Stream-based Active Learning is feasible and label-efficient for continuous deployment. Gradient-based Active Learning combined with an AAE for feature normalization improves model generalization and sample selection, supporting a practical path toward continuously learning for surgical AI systems (Code).

## Introduction

AI models have become better and better at understanding surgical scenes and solving tasks such as instrument detection [1] and segmentation [2], phase recognition [1, 3] or action recognition. Current research in surgical AI often focuses on achieving high performance on fixed, retrospectively collected datasets, with model deployment and maintenance receiving little attention. Most models are treated as static entities once in the OR, with little consideration of how they might improve, adapt, or interact with clinical experts post-deployment.

In contrast, the broader AI community is already aware of operational challenges and ways to address them [4]. Other fields that utilize AI, such as autonomous driving, fraud detection or process monitoring, commonly adopt continuous model life cycles including targeted data collection and retraining [5]. Similar approaches could benefit surgical AI systems, enabling them to improve over time and adapt to changing surgical environments.

In our view, surgical AI systems should be designed to incorporate expert feedback dynamically. When they are wrong or express low confidence, surgeons, who are the domain experts and primary users, should be able to provide corrective annotations from which the system can learn. While recent work on Test-Time Adaption [6] allows temporary, per-case adjustments, these adaptions typically do not result in lasting improvement. In contrast, a framework combining continuous data annotation and retraining could enable long-term model refinement.

However, annotation in the OR is inherently challenging: time pressure and cognitive load limit the amount of user feedback that can be provided. Label-efficient annotation strategies, which are strategies that need fewer annotations to achieve the same prediction performance, are therefore essential. Active Learning (AL) [7] addresses this challenge by identifying the most informative samples for annotation. The most commonly studied setting is Pool-based AL, where all unlabeled data are available upfront. However, in an online surgical setting, data arrives sequentially as the surgery progresses. This motivates the use of Stream-based AL, where the model must decide online whether to query an expert for annotation before the next frame arrives, without access to the whole data distribution.

Moreover, surgical scenes vary widely across institutions, surgeons, and patients. Models trained on a single dataset often fail to generalize due to distribution shifts in anatomy, tools, and lighting conditions. Continuous data collection and adaptive annotation strategies can mitigate this by targeting uncertain and diverse samples that enhance generalization.

In this work, we explore continuous data collection and Active Learning in the surgical domain. We aim to leverage the expert’s online feedback, prioritizing queries on images immediately after they are encountered, rather than delaying annotation. We adapt a gradient-based, stream-oriented Active Learning method (VeSSAL [8]) to operate under intraoperative conditions, introducing an adversarial feature normalization term to enhance diversity. Furthermore, we identify and analyze theoretical shortcomings in the original VeSSAL formulation and propose corrections that lead to more stable and label-efficient querying behavior. Motivated by the need for efficient annotation in the OR, we focus on binary classification tasks, which could be answered by experts with a single yes/no query.

Our contributions are threefold:

1. We demonstrate the feasibility of gradient- and stream-based Active Learning for intraoperative surgical AI, comparing against conventional uncertainty-based methods and equidistant sampling.
2. We show that incorporating an Adversarial Autoencoder (AAE) loss for feature normalization improves the generalization properties of the trained models.
3. We identify theoretical inconsistencies in the original VeSSAL approach, provide a principled correction, and show that this modification increases label efficiency and sampling stability in combination with the AAE in surgical data streams.

## Related work

**Active Learning** seeks to maximize model performance given limited annotation resources [7]. The underlying assumption is that given a large, unlabeled dataset, there is a subset of samples which provides the greatest improvement to model generalization if labels were available. The main challenge is then to identify high value samples and approximate the optimal subset, given an initial model trained on a fraction of the data. This subset is then presented to an expert or *oracle*, who annotates the data, then the model is retrained. This can be repeated until annotation resources are spent or model performance is acceptable. AL methods differ primarily in how they estimate the sample informativeness as well as the scenario in which the data are available.

The most common scenario is *Pool-based Active Learning*, where all unlabeled data are available simultaneously. This has been widely explored in the context of surgical AI, including for instrument and structure segmentation [2, 9] as well as workflow recognition [3]. Recent prospective studies have demonstrated the practical feasibility of AL in clinical workflows. [10] investigates pool-based AL for surgomic feature extraction for robot-assisted minimally invasive esophagectomies (RAMIE), performing prospective offline annotation and evaluating models live in the OR. Their study highlights how annotation and model evaluation can be integrated into surgical practice, an approach that could extend toward continuous data collection and model improvement, as pursued in this work.

In contrast, *Stream-based Active Learning* assumes that data arrives sequentially, requiring the model to decide immediately whether to query an annotation before the next sample appears. This setting has been widely adopted in dynamic industrial applications such as process monitoring, anomaly detection, and autonomous driving [5], where data streams are high-velocity and labeling is costly. To the best of our knowledge, such strategies have not yet been explored in the surgical domain.

Common acquisition functions for AL rely on model uncertainty, sample diversity, expected model change or variance reduction [7]. Gradient-based methods such as BADGE [11] and its streaming variant VeSSAL [8] aim to combine uncertainty and diversity, by estimating a lower bound on the model gradient if a label were available. VeSSAL is specifically motivated by a setting where the size of the dataset is unknown and the labeling budget is defined by an ideal labeling rate *q*, which should be equitably distributed across the stream.

**Fig. 1:**
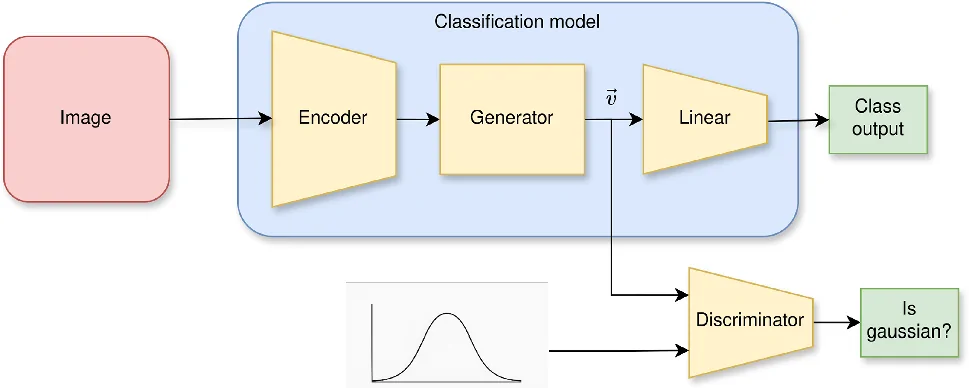
Model structure including generator and discriminator to encourage normally distributed feature representations

In the surgical context, [2] applies BADGE to segmentation, while [3, 9] use uncertainty and variance-based selection. [10] uses a variance approach, and addresses temporal redundancy by introducing temporal “safe zones” around selected frames, preventing repeated sampling of near-identical images. Recent work [12] demonstrates that combining uncertainty and diversity yields superior performance across surgical vision tasks, confirming our motivation for the gradient-based stream approach.

Similar to the use case discussed in this paper, **Continual Learning (CL)** [13] also looks at adapting models to new data, specifically focusing on learning new tasks while keeping steady performance in previously learned tasks. A core assumption of CL is that no data or a limited amount of data from previous training iterations is available. This does not apply in the use case discussed in this work, our aim is to continuously grow a diverse dataset for a single task over time. CL does also not look into picking new data and actively asking for expert input.

**Adversarial Autoencoders** [14] regularize the latent feature distribution by introducing a discriminator that enforces a target prior, often Gaussian. This adversarial alignment encourages stochasticity in the input data to be reflected in the feature representation. Spectral normalization [15] is commonly applied to stabilize the discriminator. In the medical domain, [16] applies AAEs for adverse event detection, showing that adversarial feature regularization can improve classification performance.

**Multi-task learning** (MTL) seeks to optimize multiple objectives simultaneously, often by combining task-specific losses with tunable weights [17]. Gradient-based approaches instead adjust task weighting dynamically to balance the influence of each loss. Early methods such as GradNorm [18] balance gradient magnitudes, while PCGrad [19] additionally considers gradient directions to reduce interference between tasks. The recent formulation by [20] provides a unified theoretical view of gradient-based MTL. The authors also introduce a practical software library supporting interchangeable MTL strategies. In our work, we build upon this framework and employ the AlignedMTL method [21], which aligns orthogonal components of task gradients to promote training stability.

## Methods

First, we define the annotation workflow considered in this work. The process is divided into two alternating phases: intraoperative and interoperative. At the start of each surgery, a model trained on all previously collected data is deployed together with an initialized AL strategy and a desired label rate *q*, representing the annotation budget (for example one query per minute). During surgery, video frames are processed at a fixed frame rate, and the AL strategy decides whether to query the expert for the label of the current frame. Queried frames and annotations are stored for later use. After the surgery, all collected samples are added to the training set, and the model is retrained before the next procedure.

During surgery, annotations are provided by a clinical expert, typically a surgeon, which may interrupt the surgical workflow. To minimize cognitive load and interaction time, we restrict our study to binary classification, enabling annotation through a single yes/no decision per queried frame. Multiple classes are handled by training independent models per class. We decide against standard multi-label formulations, as they require multiple annotations per image, with annotation effort increasing linearly with the number of classes. Thus, we trade off computational scalability for annotation scalability.

Next, we present the basics of the VeSSAL method as well the theory of our changes to the method. We mostly follow the math of the original and only note here where we deviate from [8].

The basic approach of both BADGE and VeSSAL is the concept of hallucinated gradients: If the current model prediction is correct, what is the gradient $g_t$ of the second-to-last layer? For the Cross Entropy loss, the analytical expression for this gradient at time *t* is given by

1 $$\begin{aligned} g_t\left( x\right) = \left( \hat{y} - p\right) \vec {v} \left( x \right) \end{aligned}$$

where $\hat{y}$ denotes the predicted label, *p* the predicted probability of that label, and $\vec {v}$ the feature representation of the input image *x* obtained from the networks encoder. This formulation inherently models uncertainty and diversity. The term $\left( \hat{y} - p\right)$ captures *prediction uncertainty*, with its magnitude increasing for uncertain samples near the decision boundary, where p≈0.5. The feature representation $\vec {v}$ encodes *semantic diversity* in the data: samples that appear dissimilar to the model are mapped to distinct regions of the feature space, leading to gradient vectors pointing in different directions. Consequently, the set of gradients $\left\{ g_t\right\}$ spans a larger volume in gradient space when both uncertainty and diversity are high. This geometric interpretation motivates selecting samples that jointly maximize uncertainty and diversity of feature representations.

VeSSAL argues that a high volume of chosen samples coincides with a large determinant of the covariance of picked samples $\Sigma _t$. For any newly encountered samples, they present it to the oracle with probability $p_t$ proportional to the contribution of the current sample to that covariance:

2 $$\begin{aligned} p_t \propto \text {det}\left( \Sigma _t + g_t(x) g_t(x)^\textrm{T} \right) \end{aligned}$$

However, they also use an uncommon definition for the covariance with ${\sum _n g\left( x \right) g\left( x \right) ^\textrm{T}}$ instead of

3 $$\begin{aligned} \Sigma _t = \frac{1}{n} \sum _n g_t(x) g_t(x)^\textrm{T} \end{aligned}$$

with the number of already picked samples *n*. If we apply the common definition of $\Sigma _t$, the VeSSAL proposition for $p_t$ is actually

4 $$\begin{aligned} p_t \propto \text {det}\left( n \cdot \Sigma _t + g_t(x) g_t(x)^\textrm{T} \right) \end{aligned}$$

This redefinition of the variance has an impact on the behavior of $p_t$. With a higher number of picked samples, over the course of an Active Learning round the relative contribution of the term $g_t(x) g_t(x)^\textrm{T}$ to $p_t$ will be smaller, so the probability of picking the current sample will be less dependent on the actual value of $g_t$. We find this an undesirable property, so we stick to the common definition of Eq. 3, while also redefining $g_t$ to adjust for the mean of all picked samples $\mu _{t}$:

5 $$\begin{aligned} \hat{g}_t(x) = g_t( x ) - \mu _{t} \end{aligned}$$

Following VeSSAL, Eq. 2 can be rewritten as

6 $$\begin{aligned} p_t&\propto \text {det}\left( \Sigma _t \right) \left( 1 + \hat{g}_t( x ) \hat{\Sigma }_t^{-1} \hat{g}_t( x )^\textrm{T} \right) \end{aligned}$$

7 $$\begin{aligned} p_t&\propto 1 + \hat{g}_t( x ) \hat{\Sigma }_t^{-1} \hat{g}_t( x )^\textrm{T} \end{aligned}$$

8 $$\begin{aligned} p_t&\propto \hat{g}_t( x ) \hat{\Sigma }_t^{-1} \hat{g}_t( x )^\textrm{T} \end{aligned}$$

where $\hat{g}_t( x ) \hat{\Sigma }_t^{-1} \hat{g}_t( x )^\textrm{T}$ is the square of the Mahalonobis distance, which describes the distance of a point from a distribution, measured in standard deviations.

However, the last step only holds true when $\hat{g}_t( x ) \hat{\Sigma }_t^{-1} \hat{g}_t( x )^\textrm{T}$ ≫1, which in general is not the case. For example, assuming a normal distribution for $g_t$, the Mahalanobis distance would be expected to be 2 or less in 95% of cases. If we implemented VeSSAL following Eq. 8, we would often underestimate $p_t$. Following our goal to achieve a stable ratio *q* of picked samples, we instead follow Eq. 7.

This change has a small impact on the derivation of the normalization factor $z_t$:

$$\begin{aligned} \mathbb {E} \left[ z_t \cdot \left( 1 + \hat{g}_t( x ) \hat{\Sigma }_t^{-1} \hat{g}_t( x )^\textrm{T} \right) \right]&= z_t \cdot \mathbb {E} \left[ 1 + \hat{g}_t( x ) \hat{\Sigma }_t^{-1} \hat{g}_t( x )^\textrm{T} \right] \\&= z_t \cdot \left( 1 + \mathbb {E} \left[ \hat{g}_t( x ) \hat{\Sigma }_t^{-1} \hat{g}_t( x )^\textrm{T} \right] \right) \\ \end{aligned}$$

And then following VeSSAL again, we end up with:

9 $$\begin{aligned} p_t = q \cdot \frac{ 1 + \hat{g}_t( x ) \hat{\Sigma }_t^{-1} \hat{g}_t( x )^\textrm{T} }{1 + \text {tr} \left( \hat{\Sigma }_t^{-1} \Sigma _a \right) } \end{aligned}$$

with our desired label rate *q* and the covariance of all encountered samples $\Sigma _a$. We estimate the covariances $\Sigma _t$ and $\Sigma _a$ in practice using an empirical estimator. We combine our changes under the name VeSSAL*.

In summary, the difference to VeSSAL lies in the addition of 1+ in numerator and denominator in Eq. 9 as well as the estimation of the covariances. Interestingly, if we follow VeSSAL and drop the 1+ factor from the derivation of the expected value, the factor *n*, by which the Covariance definition is off, is cancelled out.

Concerning the AL baseline strategies, we implement the least confidence criterion [7] for uncertainty sampling, and a variance-based strategy following [10], sampling the model 10 times for every image with a dropout rate of 0.2.

**Fig. 2:**
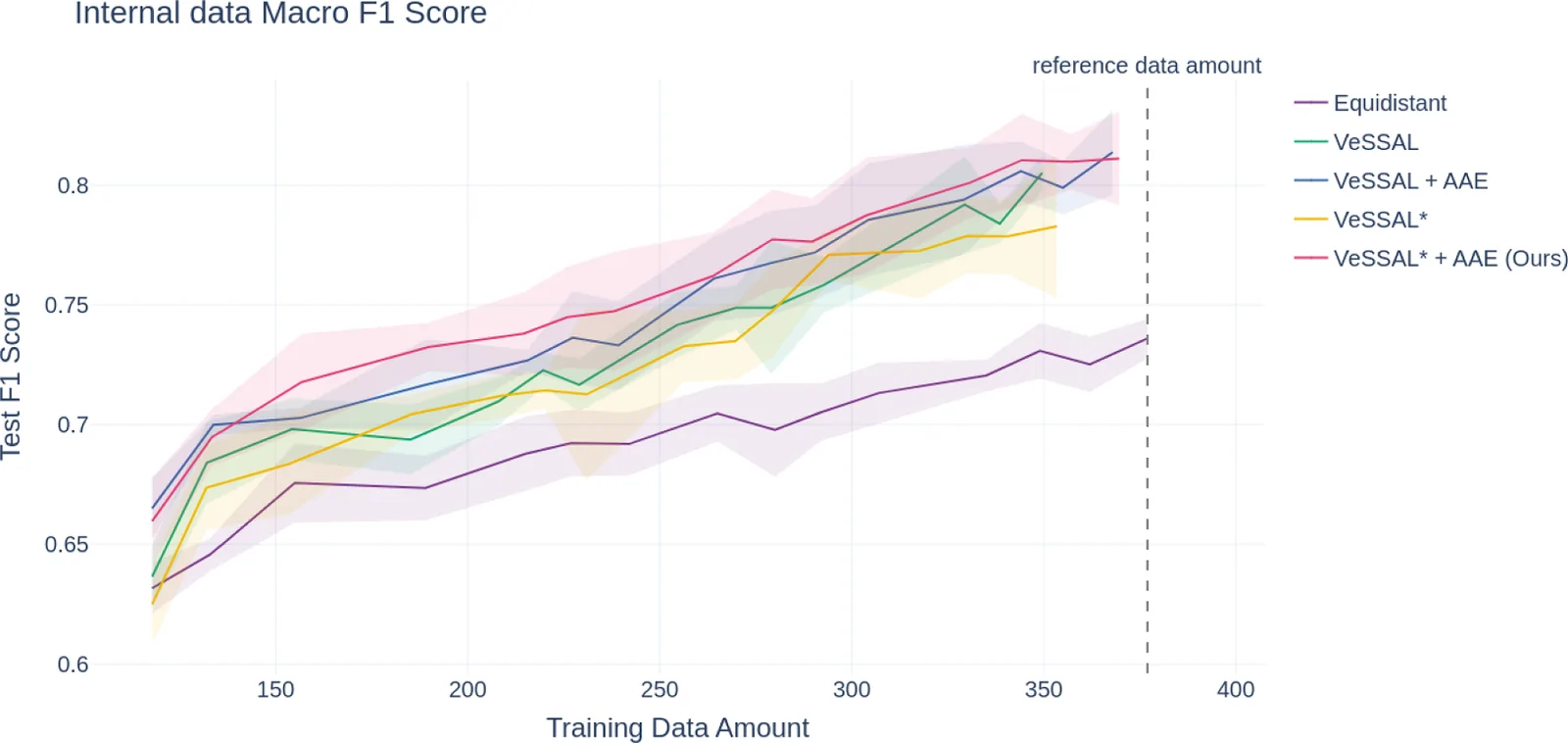
Ablation of AAE and VeSSAL* contributions. Our approach performs best on most training set sizes. In the experiments without AAE, VeSSAL* outperforms VeSSAL only on three training set sizes

Each AL method requires initial statistical estimates about the dataset, which we estimate from the validation set. For the uncertainty and variance-based strategies we determine the threshold *T* above which a sample is chosen as the percentile of data given by our target label rate *q*. For the VeSSAL* strategy, we estimate the covariance $\Sigma _a$. Importantly, these statistics do not require labeled data and could be computed from any data that the model has not seen during training, as long as it is from the same distribution (i.e., same surgery type).

As our proposed AL strategy utilizes feature diversity, we aim for a well-structured feature space, so we extend our encoder with an Adversarial Autoencoder [14], as can be seen in Fig. 1. The AAE serves as a feature regularizer, aligning the latent distribution of the encoder to a Gaussian prior.

Our AAE module consists of a pretrained encoder followed by a three-layer MLP acting as a generator. The generator preserves the latent dimensionality and produces a normalized feature vector $\vec {v}$, which is constrained by an adversarial loss to follow the Gaussian prior. A discriminator network, also consisting of 3 MLP layers, distinguishes between generated feature vectors and true Gaussian samples, and the generator is trained to fool this discriminator. The total loss combines the classification loss and the AAE loss using AlignedMTL [21] with equal weights. This formulation ensures that feature normalization supports, rather than competes with, classification performance.

## Experiments

### Datasets

We evaluate our approach on two datasets: a private dataset and the public Cholec80 dataset [1]. For both datasets, we initialize a model on one surgery, then start Active Learning with a desired label rate *q*, reflecting the allocated annotation budget. Per round, we present the model with a video stream of one surgery, then retrain the model. Because of limited compute resources, we limit our analysis to 6 labels per dataset. For both datasets, we run AL experiments until reaching 15 surgeries.

The internal dataset follows the annotation protocol of [10]. It comprises 25 RAMIEs performed at one center and annotated by one medical expert, sampled with a frequency of 1 frame per 2 min. The average surgery duration is 4 h and 32 min. We split the data into 15 / 5 / 5 surgeries for training, validation and testing. We focus our analysis on six binary labels: Azygos Vein, Gastric Tube, Vessel Sealer, Permanent Cautery Hook, Monopolar Curved Scissors and Suction. The least represented of these is the Monopolar Curved Scissors with a prevalence of 2.8%.

Active Learning is applied with $q=\frac{1}{8}$, corresponding to one query every 16 min on average. The initial training set consists of one surgery without downsampling. It contains a single positive example for the label Monopolar Curved Scissors.

Cholec80 consists of 80 laparoscopic cholecystectomy videos annotated with surgical phases and instrument presence labels. We reuse the data splits from [3], using 2 splits for training, and one each for validation and test. We focus on these labels: Bipolar, Hook, Scissors, Clipper, Irrigator and Specimen Bag.

**Fig. 3:**
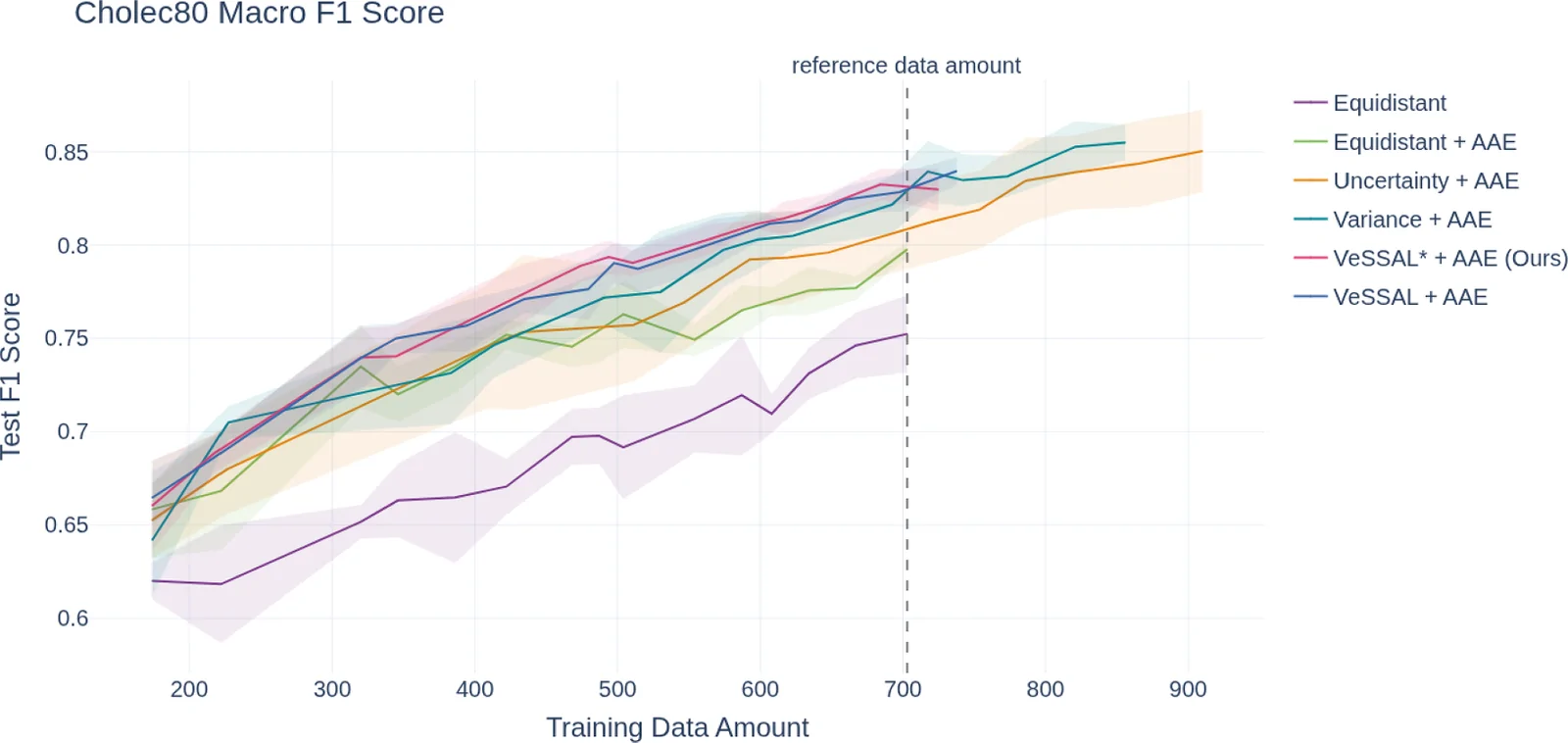
Evaluation of prediction performance on Cholec80

The initial training set consists of one surgery, downsampled by a factor of 10. During AL, we sample with $q=\frac{1}{60}$, corresponding to one query per minute.

### Implementation details

We use a ConvNeXtV2-nano backbone [22] pretrained on ImageNet. We reinitialize the model after every surgery, training with a batch size of 8/32 for internal data/Cholec80, weight decay of $1\times 10^{-3}$, label smoothing of 0.1, and dropout of 0.2. Data augmentation is performed using AugMix [23] with severity 1. We estimate optimal learning rates using an LR range test [24]. Training follows a linear learning rate warmup for the first 20 epochs, reaching $1\times 10^{-5}$ for the internal dataset and $3\times 10^{-5}$ for Cholec80. After warmup, the learning rate is halved every 10 epochs, until reaching 70 epochs. We use the Adan [25] optimizer with the cautious [26] modification. For our classification loss, we employ Focal Loss in the uncertainty-calibrated variant of [27].

The AAE module is trained jointly with the classifier. The discriminator is trained by masking 50% of its inputs to improve robustness and force the discriminator to focus on multiple features. For every generator update, the discriminator is updated 10 times to maintain equilibrium. Compared to [16], we do not require a layer freezing scheme and always train the complete model. We found that the inclusion of AlignedMTL is the most important step for achieving stable training with the AAE.

### Evaluation

We report macro averaged F1 score as our main evaluation metric. All experiments are repeated five times and are trained as binary classification tasks (one classifier per label), as discussed before. To assess how well the different strategies adhere to a given annotation budget, we also calculate the *labeling ratio* as the ratio $\frac{q_a}{q}$ of the achieved label rate $q_a$ and the desired rate *q* for each AL iteration. We report this ratio for all experiments in Table 1.

We perform ablations of our contributions for the inclusion of AAE and improvements to VeSSAL on the internal dataset, as shown in Fig. 2. All gradient-based AL approaches we investigate show increased prediction performance over the equidistant baseline, our method combining VeSSAL* and AAE provides an increase in F1 Score of 7.5% after 15 surgeries. Most of this improvement can be attributed to the label “Monopolar curved scissors”, which improves by 23% from 50%. The final performance of equidistant sampling is achieved by our method after the 5th surgery. None of the methods sample more than the desired label rate, the largest outlier being standard VeSSAL, achieving an average labeling ratio of 0.89±0.18, while our method achieves 0.97±0.15.

We then evaluate the gradient-based methods against uncertainty and variance-based sampling on Cholec80, the results are portrayed in Fig. 3. Both uncertainty and variance-based methods on average query for labels more often than allocated, with average labeling ratios of 1.31±0.67 and 1.23±0.62, respectively. Gradient-based approaches slightly exceed the desired label rate. For dataset sizes within the given annotation budget, gradient-based approaches achieve an increased F1 score after the third surgery over all baselines. VeSSAL* slightly improves over VeSSAL, ending up with an improvement of 3.2%/7.7% after 15 surgeries over the equidistant approach with/without AAE. Our method specifically outperforms the equidistant strategy on labels “Clipper”, “Irrigator” and “Specimen Bag” by 15.3%, 12.4% and 7.1%, respectively. The F1 score of equidistant sampling with/without AAE is outperformed after 10/5 surgeries by our approach.

## Discussion

Our results show that the proposed VeSSAL* strategy with AAE regularization improves label efficiency and sampling stability compared to the original VeSSAL, uncertainty-, variance-based, and equidistant sampling. Across both evaluated datasets, VeSSAL* consistently adheres more closely to the allocated annotation budget while achieving higher F1 scores.

The largest performance gain is observed for underrepresented classes. This suggests that the combination of feature normalization and gradient sampling enhances the model’s ability to identify informative samples in sparsely represented regions of the feature space.

**Table 1:** Mean labeling ratio and standard deviation, averaged over all classes and surgeries

	Internal data	Cholec80
Equidistant (reference)	1.0±0.00	1.0±0.00
Uncertainty + AAE	–	1.31±0.67
Variance + AAE	–	1.23±0.62
VeSSAL	0.89±0.18	–
VeSSAL*	0.91±0.14	–
VeSSAL + AAE	0.96±0.14	1.04±0.13
VeSSAL* + AAE (Ours)	0.97±0.15	1.02±0.12

Values close to 1 with low standard deviation indicate stable adherence to the given annotation budget

On higher frame rate data, VeSSAL* maintains stable labeling ratios, whereas uncertainty and variance-based approaches frequently sample more than they are instructed to. This confirms that gradient-based metrics are more robust to redundancy in highly correlated surgical video streams.

Despite these promising results, three main limitations remain. First, our experiments are restricted to binary classification for each label, simplifying the annotation process but limiting scalability as the number of surgomic features increases. Future work should investigate training strategies for multi-label and partially annotated datasets to support more complex and heterogeneous labeling scenarios. Second, while our approach enables online data selection, the feedback loop between expert and AI model remains slow, as model updates occur only between surgeries. Integrating Test-Time Adaptation could enable immediate adaptation to new annotations, further enhancing system responsiveness.

Lastly, the adversarial training component of our method introduces additional design complexity, including sensitivity to architectural and optimization choices. Very recent work [28] proposes a differentiable test statistic (SigReg) to enforce Gaussian structure in feature representations. This follows the same objective as our work, but achieves it with a potentially more stable method without adversarial components. Future work could explore whether this proposed loss can function as a drop-in replacement to our discriminator network.

In summary, our study provides evidence that gradient-driven, stream-based Active Learning with feature normalization offers a viable path toward continuously improving surgical AI systems under realistic clinical constraints.
